# Sb(V) Reactivity with Human Blood Components: Redox Effects

**DOI:** 10.1371/journal.pone.0114796

**Published:** 2015-01-23

**Authors:** Silvana López, Luis Aguilar, Luis Mercado, Manuel Bravo, Waldo Quiroz

**Affiliations:** 1 Instituto de Química, Pontificia Universidad Católica de Valparaíso, Valparaíso, Chile; 2 Instituto de Biología, Pontificia Universidad Católica de Valparaíso, Valparaíso, Chile; University of Alberta, CANADA

## Abstract

We assessed the reactivity of Sb(V) in human blood. Sb(V) reactivity was determined using an HPLC-HG-AFS hyphenated system. Sb(V) was partially reduced to Sb(III) in blood incubation experiments; however, Sb(III) was a highly unstable species. The addition of 0.1 mol L^−1^ EDTA prevented Sb(III) oxidation, thus enabling the detection of the reduction of Sb(V) to Sb(III). The transformation of Sb(V) to Sb(III) in human whole blood was assessed because the reduction of Sb(V) in human blood may likely generate redox side effects. Our results indicate that glutathione was the reducing agent in this reaction and that Sb(V) significantly decreased the GSH/GSSG ratio from 0.32±0.09 to 0.07±0.03. Moreover, the presence of 200 ng mL^−1^ of Sb(V) increased the activity of superoxide dismutase from 4.4±0.1 to 7.0±0.4 U mL^−1^ and decreased the activity of glutathione peroxidase from 62±1 to 34±2 nmol min^−1^ mL^−1^.

## Introduction

Automobile and heavy vehicle brake pad systems use Sb_2_S_3_ as a lubricant. Uexküll et al. suggested that the heat produced during braking may transform a portion of non-toxic Sb_2_S_3_ into Sb_2_O_3_, a compound that is classified as possibly carcinogenic to humans by the IARC (International Agency for Research on Cancer) [1]. Jang et al. showed that this reaction occurs at temperatures close to 380°C, which is easily attainable during the braking process of heavy vehicles [2].

Thus, Sb is now directly associated with vehicular traffic. In Tokyo, a survey of Sb concentrations in particulate matter conducted from 1994 to 2004 found that particles less than 2 μm in size contained 199 μg g^−1^ of Sb; furthermore, enrichment factors for these particles reached 20,900, making Sb one of the most enriched elements in this matrix [3]. Similar enrichment factors for PM2.5 between 10,000 and 20,000 were found in different cities in the USA [4].

Sb can enter an organism via trophic or non-trophic mechanisms. There are three main non-trophic sources of exposure to Sb for humans, including exposure for occupational reasons, therapeutic uses and environmental exposure in large cities. Over the last 150 years, mine workers and Sb processing plant employees have been exposed to high levels of Sb, generally in the form of Sb sulfates and oxides (Sb_2_S_3_ and Sb_2_O_3_, respectively) [5].

Our research group previously reported Sb enrichment in the urine of people exposed to contamination at mining sites [6] and in the blood of people exposed to heavy vehicular traffic in the city of Valparaiso, Chile [7]. A similar enrichment was found in urban dust and particulate matter from the same area [8]. In addition, we previously evaluated the distribution of Sb(V) in human blood fractions. These data showed that Sb(V) entered erythrocytes using time-dependent and dynamic entry and excretion processes [9].

From an environmental point of view, inorganic Sb(V) is considered to be less toxic than inorganic Sb(III) [10].

There are no published studies on the reactivity of inorganic Sb(V) in the blood. However, a few studies that evaluated the reactivity of Sb(V) in other cell systems are reported in the scientific literature.

For example, Yan et al. (2003) reported that stibogluconate, a pentavalent antimony compound used for the treatment of leishmaniasis for decades, was produced from the reduction of organic Sb(V) by trypanothione (TSH), a redox active peptide commonly found in Leishmania parasites. Sb(V) reduction by TSH was investigated at different temperatures and pH values. The results showed that Sb(V) was reduced under both mildly acidic (pH 6.4) and neutral conditions (pH 7.4) at 310 K and occurred 200 times faster than the reduction of GSH [11]. In contrast, Sb(III) binds to trypanothione at the two thiolates of the cysteine residues, which can produce a Sb(III)-(TS_2_) binary complex with a stability constant of log K = 23.6 or a ternary complex with an additional GSH molecule (GS)-Sb(III)-(TS_2_) [12].

Leishmanial As(V) reductase (LmACR2) is a member of the third molecular family of As reductases found in eukaryotes. This enzyme was recently isolated, and its structure was characterized [13,14]. Zhou et al. proposed an LmACR2-dependent mechanism for the reduction of organic Sb(V) compounds used for the treatment of Leishmaniasis. These authors demonstrated that LmACR2 can reduce As(V) and the organic Sb(V) compound Pentostam. Transfection of the *Leishmania infantum* parasite with LmACR2 augmented the sensitivity of intracellular amastigotes to Pentostam. The proposed action of Pentostam in macrophage-associated amastigotes of Leishmania involves two possible reduction mechanisms. In one pathway, Pentostam is taken up by macrophages and a portion of the compound is reduced to Sb(III), which is then transported into the amastigote through the AQP1 channel. In another pathway, the Pentostam is taken into the amastigote and reduced to Sb(III) by LmACR2 [15]. Another enzyme likely involved in the reduction of Sb(V) is the thiol-dependent reductase (TDR1), which uses glutathione as the reductant [16]. Overall, the organic compounds of Sb(V) used for the treatment of Leishmaniasis act as pro-drugs that are reduced to form more reactive Sb(III) compounds. Some of the bioinorganic effects of Sb on cells are unknown. For example, low molecular weight (LMW) thiols, such as glutathione, have been shown to reduce organic Sb(V); however, this process is too slow to be biologically significant. Despite the fact that Sb(III) binds strongly to thiolate sulfurs, these complexes are kinetically labile toward thiolate ligands, such as glutathione and trypanothione. Thus, it has been proposed that a Sb(III)-(TS_2_) complex may be extruded from the cell by the As pump, although direct evidence is needed to verify this hypothesis [17].

Determination of the reactivity of organic Sb(V) compounds used for the treatment of Leishmaniasis in cell systems has been of great interest. However, inorganic Sb(V) reactivity in red blood cells cannot be explained from these studies for two main reasons:

1. Pentostam is an organic Sb(V) compound that is soluble, but it is less polar than inorganic Sb(V), which is present as an inorganic Sb(OH)_6_− anion in solution.
2. These studies focused on Leishmania, a nucleated cellular system in which the peptide trypanothione is predominant. In contrast, erythrocytes are anucleated, and glutathione is the main peptide used to control redox balance.

The majority of redox chemistry in the blood is controlled by the glutathione redox couple GSH/GSSG, which has a redox potential of −0.24 V (25°C, pH = 7) [18]. In contrast, the inorganic Sb(V)/Sb(III) redox couple has a redox potential of approximately 0.1 V at the same conditions [19]. Thus, inorganic Sb(V) should theoretically be reduced to Sb(III) in human blood due to the presence of the glutathione produced from a redox imbalance.

Considering that Sb(V) is the most abundant form of Sb in nature, it is important to determine how Sb(V) reacts in human blood. Thus, the goal of our current study was to answer this question.

## Experimental

### Apparatus

The chromatographic separation of Sb species was performed using a Jasco (Easton, MD, USA) HPLC system PU-2089S Plus model equipped with quaternary pumps, degassers, an auto sampler, an injector with a 100 μL loop and a short Hamilton PRP-X100 column (100 mm × 4.1 mm) with a small particle size (5 μm).

To determine the Sb concentrations using hydride generation-atomic fluorescence spectrometry (HG-AFS), a PSA Analytical Millennium model (10055) atomic fluorescence spectrometer was used (Orpington, Kent, UK). This instrument has a continuous flow system for hydride generation coupled to a commercial dryer membrane (Perma Pure product, dryer model MD-110-12 FP) and fluorescence spectrometer. Stibine was purged with an atomization flame under an argon flow. H_2_ was produced during the hydride generation reaction between NaBH_4_ and HCl and sustained the flame. In addition, a supplementary flow of H_2_ was injected to maintain a stable argon/hydrogen diffusion flame.

The instrument was equipped with a Sb-boosted-discharge hollow cathode lamp (BDHCL) from Photron PTY Ltd. (Victoria, Australia) that was operated at 18.3 mA. Data acquisition was performed using a microcomputer with Avalon software from PS Analytical.

### Standard solutions and reagents

The purities of all chemicals and reagents used in this study were of analytical grade or higher. Deionized water (18.2 M Ω cm^−1^) was obtained using a Nanopure system (Barnstead, Dubuque, IA, USA). Glass and polyethylene containers were cleaned by soaking for 1 day in 10% v/v nitric acid (analytical grade). These containers were then rinsed several times with deionized water before use. Nitric acid (65% w/v, super pure grade) was purchased from Merck in Darmstadt, Germany.

Individual Sb(V) stock solutions were prepared from potassium hexahydroxoantimonate (KSb(OH)_6_, 99.95%). Sb(V) stock solutions (100 mg L^−1^) were prepared by dissolving an appropriate amount of the respective compounds in deionized water. These solutions were then stored in the dark at 4°C until use. Lower concentrations of Sb standards were prepared daily by diluting the stock solutions with deionized water.

### Sample collection

Venous blood samples were obtained from healthy university students (approximately 20 mL from each donor). A trained nurse used syringes to draw the blood, which was drained into plastic tubes and carefully mixed with a heparin anticoagulant in accordance with previously established methods.

The blood donors in this study consisted of 5 healthy men and women between 20–25 years of age. Every volunteer donated 6 tubes, each consisting of 5 mL of blood. To establish the effects on different blood types, control and test samples from the same donor were used for each biochemical parameter quantified.

All participating students gave their written informed consent to voluntarily donate blood for this study. The ethics committee of the “Pontificia Universidad Católica de Valparaíso” University approved this study as part of the project entitled “Antimony impact in urbane zones of Chile”. The Ethics committee determined that this study met the ethical standards of our university and the standards defined in the declaration of human rights and bioethics, UNESCO 2005. In addition, this committee ruled that this study does not violate the dignity of participants, nor does it represent a threat of moral or emotional harm.

### Blood fractionation procedures

To separate plasma and red blood cells (RBCs), approximately 4.0 mL of blood was separated in vacutainer tubes. The blood sample was centrifuged at 1000 × g for 10 min at 4°C in graduated centrifuge tubes using a swing-out rotor centrifuge to obtain RBCs. The plasma and buffy coat (the layer of white blood cells over the RBCs) were removed, and the RBCs were washed 3 times by centrifugation at 600 × g for 10 min at 4°C. Next, RBCs were re-suspended in approximately 8 mL of 5 mmol L^−1^ isotonic phosphate buffer at pH 7.4.

To obtain the erythrocyte membrane and the cytoplasmic fractions, the washed RBCs were lysed by adding approximately 8 mL of hypotonic phosphate buffer at pH 7.4 (at 4°C) under constant stirring. After the mixture was cooled on ice for 30 min, the lysed RBC membranes and the cytoplasmic fraction were separated by centrifugation at 20,000 × g for 10 min at 2°C. The supernatant was reserved for determining the Sb species concentration in the cytoplasmic fraction. Detailed blood fractionation procedures have been previously described in the literature [9].

### Sb(V) and Sb(III) determination in blood fractions

The chromatographic conditions for the separation of Sb species are summarized in Table 1. To prepare the blood fractions, 3 mL samples were used for both the cytoplasm and the plasma. First, 900 μL of 0.1 mol L^−1^ EDTA was added to the samples. Next, 3 mL of a saturated (NH_4_)_2_SO_4_ solution was added to precipitate the proteins. The samples were then mixed in 50 mL falcon tubes, and the resulting solution was centrifuged at 5000 rpm for 45 min until the supernatant was completely clear.

**Table 1:** Summary of conditions for HPLC coupled to a HG-AFS detection system for the determination of Sb(V) and Sb(III).

**HPLC (Jasco HPLC system PU-2089S Plus)**	
**Column**	Hamilton PRP-X-100 (100 × 4.1 mm id, particle size 5 μm)
**EDTA Mobile phase (mmol L^−1^)**	20, pH = 4.5
**Flow rate (mL min^−1^)**	1.5
**Injection volume (μL)**	100

**Detection HG-AFS (PS Analytical Ltd, Millennium Excalibur system)**	
**Sb BDHCL**	
**Primary current(mA)**	15
**Boosted (mA)**	18
**HCl (mol L^−1^)**	1.5
**HCl Flow (mL min^−1^)**	9
**NaBH_4_ (% w/v)**	0.75 (in 0.2% NaOH)
**Flow NaBH_4_(mL min^−1^)**	0.35
**Argon flow (primary) (mL min^−1^)**	300
**(secondary) (mL min^−1^)**	20
**Hydrogen (auxiliary) (mL min^−1^)**	60

The supernatant was cleaned by elution through a C18 cartridge. To quantitatively recover the Sb retained in the cartridge, 3 mL of EDTA was added as the mobile phase. A 1 mL aliquot of the resulting solution was collected, filtered using a 0.2 μm filter and then re-injected into the HPLC-HG-AFS system. Additional details of the cytoplasm and plasma purification procedures for Sb(V) and Sb(III) determination were previously reported [9]. Two chromatographic conditions were considered for use in this study. The first consisted of a gradient elution between EDTA and (NH_4_)_2_HPO_4_ for separating the inorganic species of Sb(V) and Sb(III) and the organic species of trimethyl-Sb(V). This method was previously described for Sb speciation in marine biota [20]. The second set of conditions consisted of an isocratic elution with EDTA to separate the inorganic species of Sb(V) and Sb(III). We have never detected the presence of TMSb(V) during our previous analyses of dozens of blood samples [9]; therefore, we chose to use isocratic elution conditions to separate and detect only inorganic species with HPLC-HG-AFS in this study. These chromatographic conditions are outlined in Table 1.

### Sb(V) reactivity experiments

To determine Sb(V) redox changes, approximately 4 mL of blood was incubated with 200 ng mL^−1^ of Sb(V) for 45 minutes. The blood was then fractionated using the same procedure as described above.

Reagent blanks were prepared to ensure a lack of Sb contamination, and 3 injections were completed for each sample to ensure reproducibility.

The Sb oxidation state in the plasma and cytoplasm was determined qualitatively using Sb(V) and Sb(III) chromatographic retention times and the chromatographic standards previously reported by our group [21].

### GSH/GSSG determination

Variations in GSH, GSSG and total glutathione (GSH/GSSG/Total) were determined using molecular fluorescence spectroscopy. The protocol provided with the Glutathione Fluorometric Assay kit was used (catalog number K264-100, BioVision). The instrument parameters of the spectrofluorometer are shown in Table 2.

**Table 2:** Operating conditions for molecular fluorescence spectrometry.

**Instrument Parameters**	
**Instrument name**	Appliskan
**Instrument version**	1437
**Instrument serial number**	2110149
**Instrument temperature(°C)**	25.1
**SW Parameters**	
**Run Software Version**	Skanlt Software 2.3 RE for Appliskan
**Current Software Version**	Skanlt Software 2.3 RE for Appliskan
**Excitation/Emission wavelength (nm)**	340/420

### Superoxide dismutase enzyme assay

The variation in superoxide dismutase (SOD) was determined using molecular absorption in the absence and presence of Sb(V) in blood plasma samples. The protocol provided for the Superoxide Dismutase (SOD) Activity Assay Kit was followed (catalog number K335-100, BioVision).

Blood plasma samples were prepared in accordance with the protocol described in the kit. The same fluorescence equipment was used in molecular absorption mode with an applied wavelength of 450 nm.

### Glutathione Peroxidase (GPx) enzyme assay

Variations in glutathione peroxidase in the absence and presence of Sb(V) was determined in blood plasma samples. The measurements were taken using a molecular absorption at 340 nm, in accordance with the protocol provided with the Glutathione Peroxidase Assay kit (Item No. 703102, Cayman Chemical).

### Statistical analysis

The error intervals for the quantitative results presented in figs. and tables were calculated at 95% confidence. To compare variances, a F-test at a 95% confidence level was used. All quantitative analyses of the GSH, GSSG, total GSH, SOD, GPx and GSH/GSSG ratios showed similar precision. To compare the statistical significance of Sb(V) on GSH, GSSG, total GSH, SOD, GPx and GSH/GSSG parameters, a two sample t-test with a 95% confidence level was used. All experiments were performed in triplicate by independently analyzing 3 tubes from each blood sample analyzing 3 tubes replicates were worked independently according to IUPAC criteria for reproducibility assays[22]. For each biochemical parameter quantified, control and test blood samples were always from the same donor.

## Results

Sb(V) concentrations of 100, 200 and 300 ng mL^−1^ were tested. The results showed that 200 ng mL^−1^ was the ideal concentration to ensure an absence of hemolysis. At higher concentrations, the erythrocytes were lysed, making it impossible to work with the plasma and cytoplasm fractions separately.

**Figure 1:**
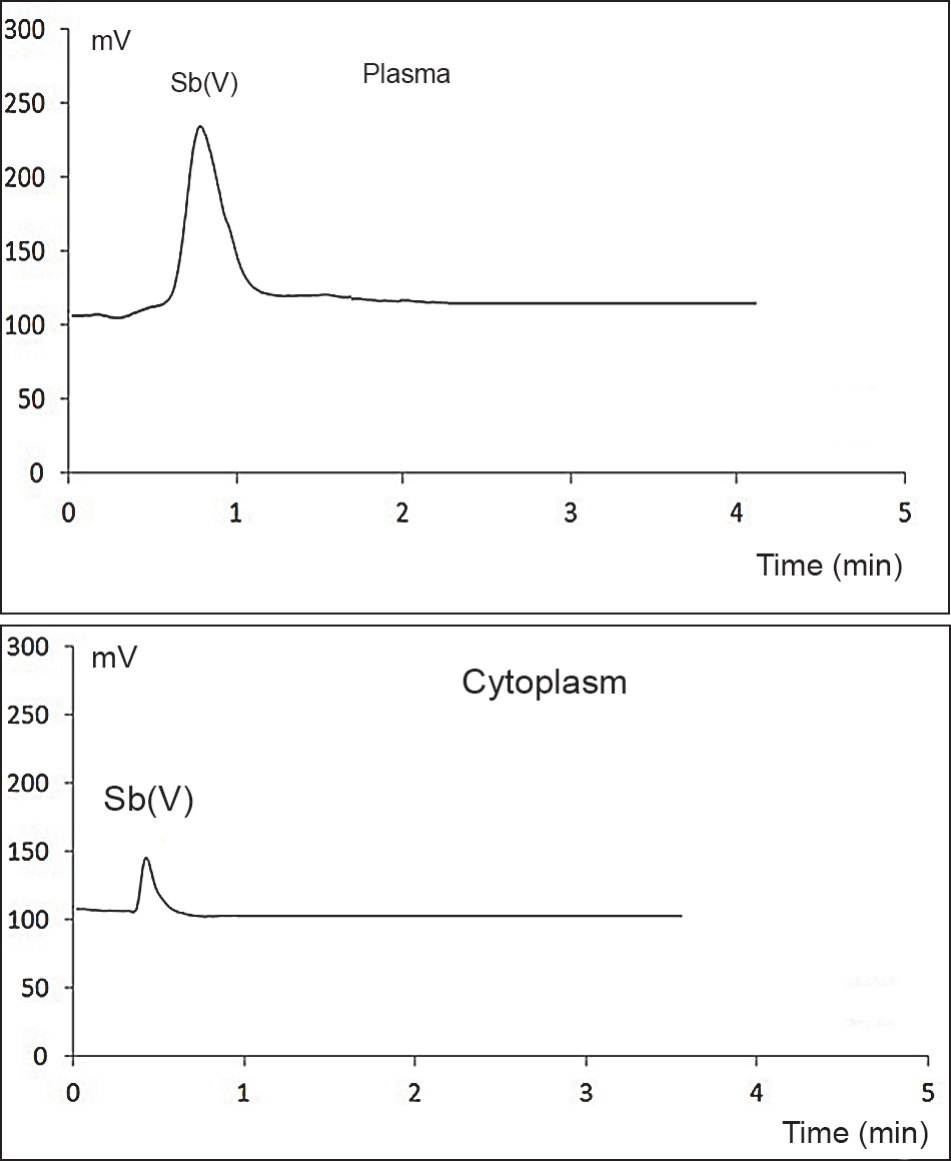
Speciation of Sb in the plasma (upper) and cytoplasm (lower) of human blood incubated with 200 ng mL^−1^ Sb(V). *No trimethyl-Sb(V) was detected. *Incubation was performed in triplicate using collection tubes from single donor’s blood.

The first experiment consisted of the incubation of triplicate blood samples with 200 ng mL^−1^ Sb(V) for 45 minutes at room temperature. The plasma and cytoplasm fractions were then separated, purified and analyzed as previously described [9]. Fig. 1 shows the chromatograms used to determine Sb(V) and Sb(III) levels in the plasma and cytoplasm.

Fig. 1 shows a clear Sb(V) signal, but no Sb(III) signal in the plasma and cytoplasm. The amount of Sb(V) in the cytoplasm was lower than in the plasma, which coincides with our previously published distribution studies [9]. The results obtained can be interpreted using two perspectives: either Sb(V) showed no redox change in any of the blood fractions or the Sb(III) formed from the reduction of Sb(V) during biochemical processes was affected by subsequent re-oxidation. Re-oxidation is likely to occur during the extended processing time required, especially for the purification of erythrocyte cytoplasm which exposes the sample to atmospheric oxygen. Therefore, reduction agents, such as GSH, may be oxidized along with the Sb(III),suggesting a general technical-based problem with the cultures.

To address the possibility of re-oxidation, Sb(III) levels were further studied. A culture was directly prepared with 200 ng mL^−1^ Sb(III) and the same sample treatment protocol was applied. The oxidation of Sb(III) to Sb(V) was observed, confirming the plausibility of this concept.

The blood was fractionated and the erythrocytes were discarded. Therefore, only blood plasma was used to avoid the hypothetical re-oxidation of Sb(III) during culturing. The plasma was purified by protein precipitation with ammonium sulfate. GSH also precipitates during this stage. Accordingly, the standard blood concentration of 5 mmol L^−1^ GSH was added in conjunction with the 200 ng mL^−1^ Sb(V). Furthermore, a control assay was conducted with plasma + GSH to remove the presence of native Sb(V) or Sb(III). In this study, these species were not detected.

**Figure 2:**
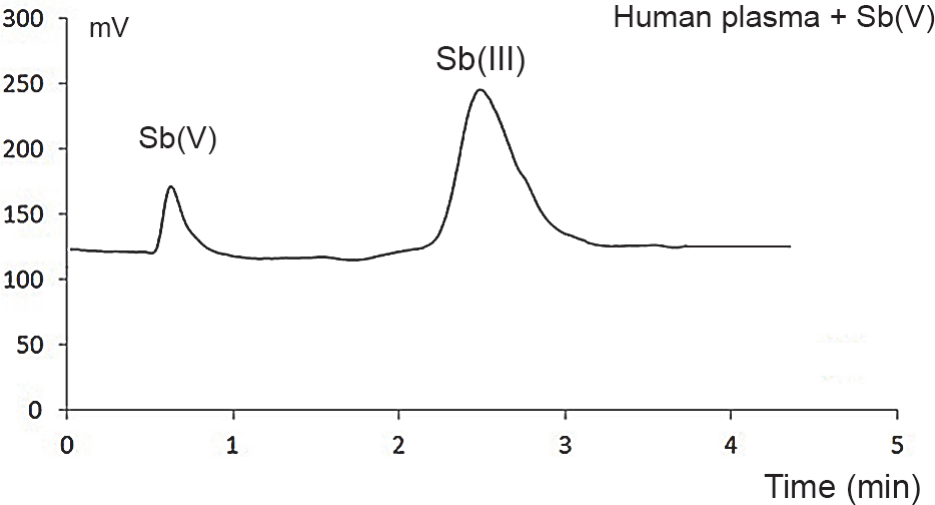
Chromatogram of blood plasma incubated with 200 ng mL^−1^ Sb(V) + 5 mmol L^−1^ GSH. *No trimethyl-Sb(V) was detected. *Incubation was performed in triplicate using collection tubes from single donor’s blood.

To minimize contact with atmospheric oxygen, the mixture time was shortened to five minutes before analysis. Fig. 2 shows the chromatogram obtained under these conditions, showing a clear reduction of Sb(V) to Sb(III) by GSH. These data coincide with the reactivity studies of organic Sb(V) compounds previously reported for Leishmaniasis [23], macrophages [24], and the redox potentials of Sb(V)/Sb(III) and GSH/GSSG couples [18,25].

These data clearly suggest that Sb(V) shows redox reactivity in the blood plasma. Thus, the results in Fig. 1 confirming the presence of Sb(V) are likely due to the re-oxidation of Sb(III) during culturing.

To stabilize Sb(III) during incubation, the stabilizing effectiveness of EDTA was evaluated. Previous studies showed that EDTA stabilized Sb(III) in multiple matrices, such as seawater [21], sediment extracts [26] and marine biota [20]. EDTA also selectively complexes with Sb(III) [27].

To prove the stabilizing capacity of EDTA, two culture media were prepared:

1. Blood plasma + 200 ng mL^−1^ Sb(III) was incubated for 45 minutes, then purified and injected.
2. Blood plasma + 200 ng mL^−1^ Sb(III) + 0.1 mol L^−1^ EDTA was incubated for 45 minutes, then purified and injected.

The results show a clear decrease in the oxidation of Sb(III) to Sb(V). In the sample without EDTA, approximately 70% of the Sb(III) was oxidized to Sb(V); however, the sample with 0.1 mol L^−1^ EDTA showed an oxidation of less than 30%. Based on these results, 0.1 mol L^−1^ EDTA was used with Sb(V) in the culture medium of blood samples.

Blood samples were incubated for 45 minutes in media containing 200 ng mL^−1^ Sb(V) and 0.1 mol L^−1^ EDTA. The blood was then fractionated in accordance with the aforementioned protocol, and the plasma was used after purification and injection into the HPLC system.

**Figure 3:**
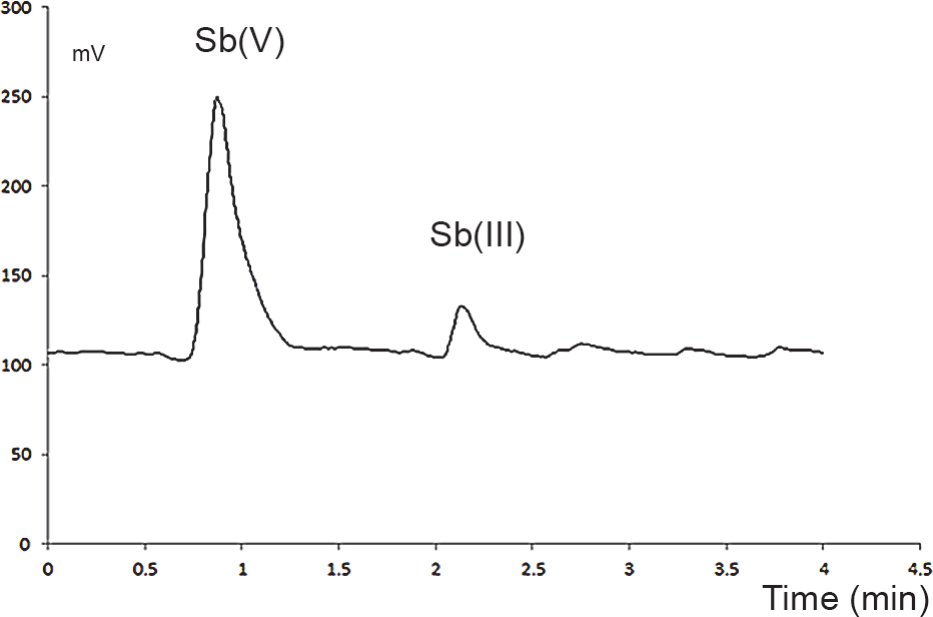
Chromatogram of blood plasma incubated with 200 ng mL^−1^ Sb(V) and 0.1 mol L^−1^ EDTA. Partial reduction of Sb(V) to Sb(III) can be observed. *No trimethyl-Sb(V) was detected. *Incubation was performed in triplicate using collection tubes from single donor’s blood.

The results in Fig. 3 show that Sb(V) was distributed in the blood plasma and a portion of this species was reduced to Sb(III), possibly using GSH as the reduction agent. These data coincide with previously reported results for the reduction of organic compounds of Sb(V) in macrophages [24] and Leishmaniasis [28].

It is important to note that the results presented in Fig. 3 suggest that the instability of Sb(III) generated during incubation and purification may require the reinterpretation of previous results related to Leishmaniasis. For example, Zhou et al. reported that the reduction of Sb(V) to Sb(III) by the LmACR2 enzyme in Leishmaniasis is below 10% [15]; thus, the consumption of Sb(V) may be underestimated due to the potential re-oxidation of newly generated Sb(III).

**Figure 4:**
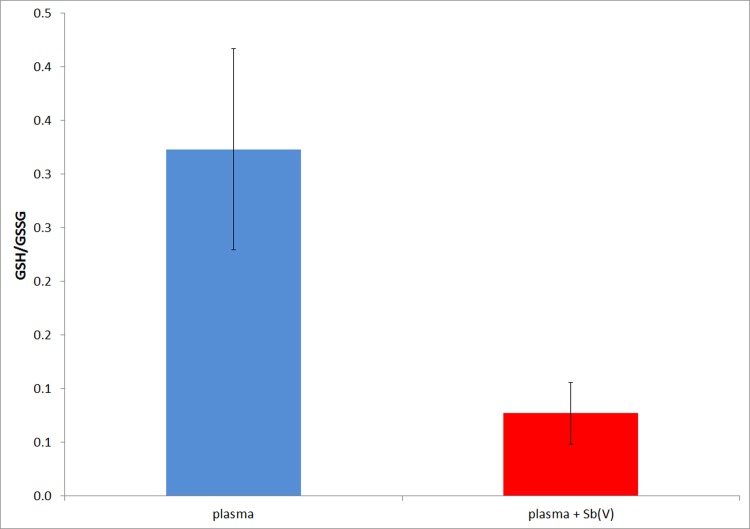
Effect of Sb(V) on the ratio of GSH/GSSG in human blood plasma. The error bars represent the standard deviations from 3 independent assays. t tests to compare data sets at 95% confidence, showed that difference is statistically significant, (t_exp_ = 4.3; t_critical; 1 tail, 4 DF_ = 2.1).

After we established the reduction of Sb(V) to Sb(III) in human blood, we next evaluated the potential reducing agents. One of the most abundant reductants in human blood is GSH. In addition to evaluating the redox transformation of Sb(V) to Sb(III), we determined the variation in free GSH and oxidized GSH (GSSG) levels in blood exposed to Sb(V). The results are presented in Fig. 4.

The results showed that Sb(V) produced a clear decrease in the GSH/GSSG ratio. These results are consistent with those shown in Fig. 3 and demonstrate that the reduction of Sb(V) to Sb(III) is directly related to the oxidation of GSH to GSSG. Notably, Sb(III) also forms a complex with GSH that is very stable though kinetically labile; thus, the decrease in the proportion of free GSH may also be related to the formation of a Sb(GSH)_3_ complex [29].

Lastly, two additional biochemical parameters for the reactivity of Sb(V) in human plasma were evaluated. The enzyme activity of superoxide dismutase (SOD) and glutathione peroxidase (GPx) was assessed. The GSH, GSSG, total glutathione (GT), SOD and GPx levels are presented in Table 3.

**Table 3:** The effect of Sb(V) on GSH, GSSG, GT, SOD and GPx levels in human plasma.

**Sample**	**GSH**	**GSSG**	**GT**	**SOD**	**GPx activity**
	**(μg mL^−1^)**			**U/mL**	**nmol min^−1^ mL^−1^**
plasma	78±15	247±39	432±12	4.4±0.1	61.5±0.9
plasma + Sb(V)	5±1	72±9	76±6	7.0±0.5	34±2
Statistical analysis					
t critical	2.8	2.8	2.8	2.8	2.8
t experimental	8.1	7.6	46.8	9.4	23.7

Confidence intervals at 95% (n = 3, all tubes were obtained from one single donor).

The results in Table 3 show that Sb(V) decreases the levels of GT and the individual species. This effect may be associated with the protein precipitation caused by the addition of the standard. Notably, the activity of SOD was significantly increased, which may be related to the increase in oxygen-reactive species caused by a decrease in the GSH/GSSG ratio as the Sb(V) reacted with GSH. The GPx results are consistent with the low levels of GSH generated by the presence of Sb(V). Taken together, these data suggest that Sb(V) generates a redox imbalance after being reduced by GSH, and thus increasing free-radical activity.

The concentrations of Sb(V) used in this study are on the order of ng mL^−1^ (μM). These concentrations are approximately 1000 times lower than the therapeutic levels of Sb(V) in Glucantime or Pentostam recommended by the World Health Organization for the treatment of Leishmaniasis, which are of the order of μg Kg weight^−1^ (mM level) [30]. Sb(V) concentrations in the mM range are typically used for studies in Leishmaniasis cells [15], including Sb(V) resistance [31] and Sb(V) reduction in Leishmania [32]. According to our results, clear redox effects are generated by Sb(V) at ng mL^−1^ levels in the blood. Thus, the side effects in the blood of patients treated with Glucantime or Pentostam at much higher Sb(V) concentrations should be addressed. However, it is important to stress that there is an important chemical difference between the chemical forms of Sb(V) in atmospheric particulate matter and Glucantime and Pentostam.

Sb(V) from atmospheric particulate matter is in an oxide form, which dissolves to form the anion Sb(OH)_6_− at neutral acidity [25]. However, the chemical form of Sb(V) in Glucantime or Pentostam in solution are larger organic anions with hydrocarbon structures that are markedly less polar [33]. These chemical differences show that reactivity and toxicity of Sb(V) in Glucantime or Pentostam is not necessarily the same as Sb(OH)_6_−. Thus, independent reactivity studies in human blood using mM levels of the organic Sb(V) found in Glucantime and Pentostam are needed.

## Conclusions

The main conclusions of this study are as follows:

Sb(V) at a concentration of 200 ng mL^−1^ showed redox reactivity in human blood. This species is reduced to Sb(III) in the presence of GSH.
Sb(III) is an unstable species in blood, and the formation of this species competes with its own redox instability when in contact with the atmosphere. Thus, the addition of stabilizing agents, such as EDTA, is mandatory for the accurate assessment of human blood.
Sb(V) redox reactivity in human blood produced an increase in free-radical activity and a decrease in the GSH/GSSG ratio.

Based on these redox effects and the fact that Sb(V) is found in APM and accumulates in blood, the actual toxicity of this species for humans exposed to vehicular traffic is of concern.

What effects arise in human blood due to the presence of inorganic Sb(III)? How does it affect soluble proteins, lipids and membrane proteins? We believe that these questions must be addressed by future research in this field. Moreover, our results highlight the need for new research perspectives on the potential side effects of Sb(V) drugs used for the treatment of Leishmaniasis on the redox chemistry of human blood.
